# Use of Mobile Apps in Heart Failure Self-management: Qualitative Study Exploring the Patient and Primary Care Clinician Perspective

**DOI:** 10.2196/33992

**Published:** 2022-04-20

**Authors:** Leticia Bezerra Giordan, Rimante Ronto, Josephine Chau, Clara Chow, Liliana Laranjo

**Affiliations:** 1 Westmead Applied Research Centre University of Sydney Sydney Australia; 2 Department of Health Systems and Populations, Faculty of Medicine, Health and Human Sciences Macquarie University Sydney Australia

**Keywords:** mobile app, mHealth, heart failure, self-management, eHealth, telehealth

## Abstract

**Background:**

Mobile apps have the potential to support patients with heart failure and facilitate disease self-management, but this area of research is recent and rapidly evolving, with inconsistent results for efficacy. So far, most of the published studies evaluated the feasibility of a specific app or assessed the quality of apps available in app stores. Research is needed to explore patients’ and clinicians’ perspectives to guide app development, evaluation, and implementation into models of care.

**Objective:**

This study aims to explore the patient and primary care clinician perspective on the facilitators and barriers to using mobile apps, as well as desired features, to support heart failure self-management.

**Methods:**

This is a qualitative phenomenological study involving face-to-face semistructured interviews. Interviews were conducted in a general practice clinic in Sydney, Australia. Eligible participants were adult patients with heart failure and health care professionals who provided care to these patients at the clinic. Patients did not need to have previous experience using heart failure mobile apps to be eligible for this study. The interviews were audio-recorded, transcribed, and analyzed using inductive thematic data analysis in NVivo 12.

**Results:**

A total of 12 participants were interviewed: 6 patients (mean age 69 [SD 7.9] years) and 6 clinicians. The interviews lasted from 25 to 45 minutes. The main facilitators to the use of apps to support heart failure self-management were communication ability, personalized feedback and education, and automated self-monitoring. Patients mentioned that chat-like features and ability to share audio-visual information can be helpful for getting support outside of clinical appointments. Clinicians considered helpful to send motivational messages to patients and ask them about signs and symptoms of heart failure decompensation. Overall, participants highlighted the importance of personalization, particularly in terms of feedback and educational content. Automated self-monitoring with wireless devices was seen to alleviate the burden of tracking measures such as weight and blood pressure. Other desired features included tools to monitor patient-reported outcomes and support patients’ mental health and well-being. The main barriers identified were the patients’ unwillingness to engage in a new strategy to manage their condition using an app, particularly in the case of low digital literacy. However, clinicians mentioned this barrier could potentially be overcome by introducing the app soon after an exacerbation, when patients might be more willing to improve their self-management and avoid rehospitalization.

**Conclusions:**

The use of mobile apps to support heart failure self-management may be facilitated by features that increase the usefulness and utility of the app, such as communication ability in-between consultations and personalized feedback. Also important is facilitating ease of use by supporting automated self-monitoring through integration with wireless devices. Future research should consider these features in the co-design and testing of heart failure mobile apps with patients and clinicians.

## Introduction

Heart failure is one of the leading causes of hospitalization, morbidity, and mortality in the world and a major public health challenge [1]. Hospitalization for patients with heart failure is well known to be associated with an increased risk of death [2,3]. Good self-management can reduce rehospitalization and exacerbations [4-7]. Therefore, it is important for patients to know how to self-manage their condition at home, monitor early signs of congestion, and take the necessary action to avoid readmission [5,6].

Heart failure self-management involves adherence to specific behaviors (eg, medication compliance) and self-monitoring (eg, weight, blood pressure, signs, and symptoms) [4,5,8,9]. Self-management can be challenging for many patients due to lack of knowledge, symptoms recognition, motivation, and ability or confidence in performing it [9-12]. Heart failure impacts patients’ lives in many ways and several physical and emotional factors may influence how patients respond to the challenges of self-management [11,13]. An analysis of self-management behavior in 15 countries showed that patients are poorly adherent to self-management tasks, and less than 50% of patients weigh themselves regularly [14]. Technology has the potential to facilitate the delivery and dissemination of self-management support and promote the ongoing surveillance and management of clinical deterioration in patients with heart failure.

Mobile technologies (eg, SMS text messaging, mobile apps, wireless monitoring devices) [15] are increasingly being used to support self-management of chronic diseases, either as part of telemonitoring programs or as stand-alone patient-facing interventions [16-18]. Interventions using mobile phone apps seem promising in cardiovascular disease self-management. Apps can automate the self-monitoring of physiological data, facilitate the tracking of symptoms, provide reminders, and offer personalized feedback to promote engagement [17,19-23].

However, in heart failure, the use of apps to support self-management is still in the early stages of research, with inconsistent results for efficacy [16]. So far, most of the published studies evaluate the feasibility of a particular app [24-31] or assess the quality of heart failure apps available in app stores [32-34]. Only a couple of qualitative studies [35,36] have analyzed users’ perspectives on supporting heart failure self-management with mobile technologies, but they did not focus specifically on mobile apps, nor did they interview clinicians. Given the growing number of feasibility studies evaluating heart failure apps, research is needed to explore the patient and clinician perspective to guide app development, evaluation, and implementation. This study aims to explore the perspectives of patients and clinicians from a primary care center in a low socioeconomic setting in Sydney, Australia, exploring facilitators and barriers to the use of mobile apps to support heart failure self-management.

## Methods

### Study Setting and Participants

This is a qualitative phenomenological study involving face-to-face semistructured interviews. This study was conducted in a single primary care practice in Western Sydney (Mount Druitt Medical Centre), Australia, providing care to a population with low socioeconomic and educational level. The clinical team comprised general practitioners and allied health professionals (eg, dietician, psychologist, counselor, clinical pharmacist, and exercise physiologist). The clinic currently utilizes an electronic health record integrated with another system consisting of a clinician web-based platform for care coordination and a free patient-facing app (Multimedia Appendix 1). The patient app was not specific for heart failure self-management but allowed patients to send messages to their health providers, input and graphically visualize health data (eg, weight, blood pressure), and receive automated feedback. The practice and sample were selected using convenience methods. Eligible participants in this study were adult patients of the clinic with a confirmed diagnosis of heart failure (regardless of its classification or stage) and health care professionals who provided care to patients with heart failure at the clinic. Participants were eligible if they were able to communicate in English. Patients did not need to have previous experience using mobile apps, nor any knowledge about heart failure mobile apps. They also did not need to be users of the clinic’s patient app to be eligible for this study. Participation in the study was voluntary and no incentive was provided.

### Participant Recruitment

Patients were contacted by the clinic via phone and provided with details about the research study. Clinicians were contacted by the clinic director and informed about the study. Those who agreed to participate in the project were then contacted by the first author (LB) to schedule a face-to-face interview at the clinic. On the interview date, the eligible patients and clinicians received the hardcopy of the consent form, having the opportunity to read it and ask any questions they may have had about the study, before providing informed consent.

### Data Collection

The interviews were conducted by LB, a Master of Public Health student, cardiologist by training, with previous experience in research and qualitative interviewing. Interview guides including demographic questions for patients and clinicians were developed and pilot tested by 2 researchers (LB and LL; Multimedia Appendices 2 and 3). The interview guides were developed based on relevant studies identified in the literature [28,37-40]. After informed consent was obtained, individual semistructured interviews were conducted and audio-recorded from February to March 2020. Field notes were taken during the interviews. Guided by the principle of thematic data saturation and existing literature indicating that a sample size ranging between 6 and 12 participants is adequate for a phenomenological study like ours, we aimed to recruit a minimum of 6 participants [41-45]. Additional individuals were recruited until the point when the researcher had the perception that no new themes or subthemes were emerging (ie, data saturation), a standard approach in qualitative research [46,47].

Patient interviews started with broad questions regarding their routine in managing their condition, challenges they faced in performing heart failure–related tasks, and the factors or strategies that could help with those tasks. Afterward, they were asked about their experience with mobile technology and the main barriers and potential facilitators to using an app for heart failure self-management support. Clinician interviews were initially focused on their usual consultations with patients with heart failure and the most important parameters they asked patients to monitor. Then, they were asked about their perspectives on the main difficulties their patients face to manage heart failure and how they thought mobile apps could potentially help patients deal with their condition.

### Data Analysis

The interviews were transcribed and analyzed using the NVivo 12 software (QRS International Pty Ltd). The interview transcripts were analyzed by 2 investigators (LB and LL) using thematic data analysis. Themes were identified using an inductive data-driven approach (ie, inductive thematic analysis) [46]. Inductive thematic analysis is a process of coding the data without trying to fit them into a pre-existing coding frame or to analytic preconceptions so that the themes identified are strongly linked to the data themselves [46]. This analysis is in contrast to theory-driven analysis (ie, theoretical data analysis), where a specific theory or theoretical approach guides the analysis [46]. First, we selected relevant information in the data, generating open codes in our codebook (first-cycle coding) [47]. As the analysis progressed, several codes were added inductively. Second-cycle coding involved focused coding (ie, to find thematic similarity) and axial coding (ie, to find relations between codes) [47]. The initial codebook was developed by LB based on 4 interviews, at which point LL and LB discussed and revised the codebook. Subsequent revisions of the codebook by the authors occurred iteratively every 3 interviews through comparing and revising codes and emerging themes. Identification of themes occurred by sorting the different codes into potential themes, and collating all the relevant coded data extracts within the identified themes. Themes were identified at a semantic level, with analysis starting by organizing data to show patterns in semantic content, and then moving to the interpretation of the patterns and their broader meanings and implications [46]. After a candidate thematic “map” was reached, the data set was re-read to ensure the quality of the themes and refine them as needed. We reached data saturation when no new themes emerged from the data.

We then compared each theme with the literature, based on a systematic review on the same topic [48], searching for common and diverse themes, refining concepts, and reviewing the major themes. Finally, we analyzed the overall information content, selected extracts for quotes, and compiled the analysis report. Reporting follows the COnsolidated criteria for REporting Qualitative research (COREQ) checklist for reporting qualitative research (Multimedia Appendix 4) [49].

### Ethics Approval

Ethical approval for this study was obtained from the Macquarie University Ethics Committee (Reference No: 52019612812569; Project ID: 6128).

## Results

### Sample Characteristics

We recruited 12 participants: 6 patients with heart failure and 6 clinicians. The patients’ age ranged from 57 to 79 years (mean 69 [SD 7.9] years) and 4 were women (Table 1). Most patients (n=5) had been diagnosed with heart failure for more than 3 years and 1 had been recently diagnosed. All participants owned a smartphone. The clinicians’ age ranged from 32 to 46 (mean 38 [SD 5.2] years), and 5/6 were women. The sample was composed of 2 general practitioners, a clinical pharmacist, a dietitian, an exercise physiologist, and a counselor. The average number of years working in the clinic was 5 years (SD 2.2).

**Table 1:** Characteristics and self-monitoring behaviors of the interviewed patients (N=6).

Characteristics							Values
**Age**							
	Mean (SD)					69 (7.9)	
	Range					57-79	
**Sex, n**							
	Women					4	
	Male					2	
**Marital status, n**							
	Single					1	
	Married					2	
	Divorced					2	
	Widowed					1	
**Occupation, n**							
		Retired				5	
		Unemployed/pensioner				1	
**Disease duration, n**							
			<1 month			1	
			3-6 years			4	
			7-10 years			1	
**Frequency of weight monitoring, n**							
				1× day		3	
				1× week/fortnight		2	
				1× month		1	
**Frequency of blood pressure/heart rate monitoring, n**							
					1× day	3	
					1-3× week	2	
					Never or seldom	1	
**Comorbidities^a^**							
					Diabetes	5	
					Hypertension	5	
					Pneumopathy	4	
					Walking impairment (arthropathy, neuropathy)	3	
					Atrial fibrillation	2	

^a^A patient can have multiple comorbidities.

### Qualitative Results

The interviews lasted from 25 to 45 minutes. Themes and subthemes emerging from the data are detailed below.

#### Facilitators to the Use of Apps to Support Heart Failure Self-management

##### Communication Features

Communication ability, particularly between patients and clinicians, was cited as one of the most helpful features in any app supporting heart failure self-management. Patients mentioned that chat-like features that facilitate communication outside of clinical appointments can be helpful for asking questions, receiving feedback, or requesting prescription renewal.

If I need to ask a question or need something, I can put it on [the app] and I know that they (clinicians) can see it, or at the Hospital. I can book appointments and speak to people.

Female, 63

Clinicians also considered helpful to send motivational messages to patients, reminding them to maintain self-monitoring, or asking them about signs and symptoms.

I think that if we have constant communication with them it would help to motivate them and keep on track.”

Clinician number 2

I would ask him to do daily weight measurements (...) But also asking them how they feel regarding shortness of breath. [...].

Clinician number 6

In addition, the ability to share audio-visual information was mentioned as helpful by patients (eg, pictures of their food) and clinicians (eg, video instructions for exercises).

##### Personalized Feedback and Education

Patients and clinicians reported that personalized feedback and educational tools were very important to improve patients’ awareness about their health status and the consequences of their behaviors.

When you have something wrong, then you have the feedback. It helps you to understand better what to do. (...) When that says ‘you've lost weight, stay on your program’. (...) I’m doing a lot more things now than what I did before the app, you know?

Male, 65

Participants said that feedback in the form of color-coded risk assessment or trend graphs were helpful features, reassuring them when they were doing well, and alerting and guiding them when they were not within the normal ranges.

You know you're doing okay or not because of the colors. Green, yellow, and red. If I’m red I’m really out. If it happens, I just get in contact with my medical advisor. Or go to the hospital. And with this [the graphs] you can check if you are doing okay, if you're on track or not.

Female, 63

Clinicians mentioned the importance of personalizing the feedback and automated messages for each patient, as they believed this contributed to improve patients’ motivation to better manage their condition.

Patients do learn from that interaction [automated feedback]. They become more able to self-manage or their family member is able to help. And they gain more knowledge of their conditions. They feel more self-sufficient or an active member (...) in health care rather than just passive. If the patient’s weight or blood pressure are all on target, they will get a good message, ‘you are on target, well done’. If they are getting a little bit out of range, the app automatically will send a message, ‘please check your weight’. If it's not improving, come and see us, and the red message will do that. And that's set by the treating team for each patient, what the message and alert levels would be.

Clinician number 3

Participants mentioned that the ability for both patients and clinicians to monitor signs of deterioration allowed for timely action.

The app creates a graph that they can see and we can see. So that is pictorial as well. And it means that we can mitigate some exacerbation.

Clinician number 4

In addition, participants indicated a desire for personalized education features (eg, daily guide for diet and liquid intake) according to patients’ age and health conditions, as well as tools to facilitate medication adherence.

I would like to have an app not only about calorie content, but more about the quality of their diet (...). It is important to increase patient awareness about what they eat. And probably more individualized, like their medication, other diseases, allergies, food intolerance.

Clinician number 1

They do get confused [...] We see patients accidently doubling up on doses because of generic and brand. It would be great if they could take a photo of the box or the barcode and it tells them if this is the same as that. That would be [an] ideal feature.

Clinician number 3

##### Automated Self-monitoring

Both patients and clinicians highlighted the helpfulness of automated self-monitoring of the main parameters in heart failure management (eg, blood pressure, weight). Patients reported that manually tracking their weight, blood pressure, and liquid intake were very burdensome tasks and that being able to automatically integrate data from devices (eg, fitness tracker) was very important.

What I find difficult to control is just everyday watching (...) the fluid I intake.

Male, 57

This doesn’t cover one or two [diseases]. You want it to cover you entirely. COPD, blood pressure. [...] I use this, Fitbit. And they can see it [steps and heart rate] in the app.

Male, 65

Professionals expressed the need for connectivity with other monitoring devices (eg, glucose meters) and the ability to automatically track different activities (eg, riding a bike) and levels of intensity.

For example, some models of watches just do step counts and heartbeat. And some can really distinguish when you're going for an exercise or a run, (...) and this information goes directly to the smartphone. Well, it can say “you're only doing 30 minutes when you need to be 45” or “you are not doing the right time, duration, and frequency.

Clinician number 2

##### Patient-Reported Measures and Mental Health Tools

Professionals considered essential to enable patients to register their symptoms and other patient-reported measures, as well as patients’ mental health and well-being. Clinicians saw mental health tools as a necessary feature to support patients in dealing with the disease burden and individual challenges. These participants suggested potential benefit in having a tool to collect and track the Patient Activation Measure [50], which measures a patient’s level of activation (ie, knowledge, skill, willingness, and confidence to perform self-management of chronic diseases). This measure was perceived as potentially helpful to understand changes over time and identify self-management determinants.

It will create self-awareness, asking the patient what they're thinking, how they are (...) feeling today. And then you can actually do something about it. [...] But I think [it is important] to be mindful that not everybody works the same. So perhaps something tailored to suit each individual (...) and it can be done through a set of questions.

Clinician number 5

If I can add one thing, is probably tools to help with mental health. Because I find a lot of patients that feel that burden.

Clinician number 1

[I would add] probably things like overall well-being score, like a mood or depression score. So they can self-evaluate how they actually feel about their health, because that is a big marker of how they will cope with all the other things.

Clinician number 3

#### Barriers to the Use of Apps to Support Heart Failure Self-management

##### Patients’ Digital Literacy and Willingness to Use Technology

Participants considered patients’ lack of digital literacy and unwillingness to use technology as important barriers to the use of mobile apps. Patients mentioned seeing themselves as not being tech-savvy enough to use an app (mostly due to a perceived age barrier) and expected it would take them a long time and effort to learn how to use it properly.

I'm not really good with phones. (...) I try to use [the clinic’s app] when my daughter is at my house. I mean, for us it's a lot harder, but I think it's a very good technology for the new generation.

Male, 57

According to professionals, patients with a stable or long-time condition would be less prone to engage in a new strategy with mobile apps to manage their condition, especially if they are not used to technology.

They [older patients] are a little bit stuck in their ways. They've sort of done things for a certain way for so long, that is hard to just [change]. In terms of the apps, they probably also are obviously not very technologically advanced.

Clinician 2

However, this barrier could be overcome by introducing the app soon after an exacerbation, when they might be more willing to improve their self-management practices.

I think when it comes to trying to motivate them, the ideal time would be when they have an exacerbation and we say “look, you don't need to come in every day while we watch this, you can get this from home, just set up with this app; this is how we're going to manage to get on a daily weight, right?”. When they are stable, they don't want to do it. But when they have an exacerbation, that would be the time, when they are discharged from the hospital.

Clinician 6

Professionals added that learning how to use an app could be an additional burden to some patients but family or caregivers’ support could be helpful.

Sometimes people go through a period where they are quite enthusiastic about their monitoring (...). Then they just lose interest. [...] They need to understand what is in there, how much information is there, input their data, send messages. I think they find that a bit overwhelming sometimes. So teaching them how to use the app is often problematic because they may do it whilst they're in the clinic and then they go home and they can't work it out again.

Clinician 4

I think it would be the combination of what else is happening in their lives. How busy are they? What family support do they have? Transport, all those all those factors (...), where's the priority? If they're busy looking [after] somebody else or kids.

Clinician 5

Time and that routine of checking it [the app] regularly [are difficult]. We always check our emails quite easily, but checking this other thing is a bit harder. [...]

Female 79

##### Clinician Workload, Remuneration, and Digital Literacy

Clinicians considered increased workload, lack of remuneration, and insufficient digital literacy as the main barriers to implementing a shared platform connected to a patient app. They said that managing patients’ data and messages was time-consuming and not remunerated but may help avoid unnecessary visits to the clinic and optimize consultations.

We don't have to text them “your blood pressure's a little bit high” or something. The app does all that stuff. There's lots of (...) things that make it less labor-intensive for the clinicians. It makes patients understand (...) “I'm stable, I'm good”, so it also allows other avenues because the ones that are stable know they don't need to come in. Then people who need quick access are able to come through the doors. And it's not just the doctors monitoring it. Our pharmacists and dieticians are part of the team, also taking responsibility. So they're actually monitoring it and helping alert us to any problems. It’s a team-based monitoring. [...] So it's definitely a little bit more work, but not to the point where you're not answering messages. [...]

Clinician 3

Professionals mentioned the importance of all clinicians being able to access and respond to patients’ data and messages, enabling them to share responsibilities and improve the team’s problem-solving ability.

It also means that we can communicate with different team members as well. [...] So we all can make suggestions, contribute and send messages to each other.

Clinician 4

#### Associations Between Barriers and Facilitators to the Use of Apps to Support Heart Failure Self-management

There was a connection between patients’ willingness to use technology and facilitators to the use of apps to support heart failure self-management. Participants mentioned the use of an app could be associated with an increase in the burden of managing heart failure. They explained that learning how to use the app and remembering to use it could be seen as an additional responsibility in people’s already busy lives, which could be demotivating and lead to a lack of interest and decreased willingness to use the app over time. Hence, participants mentioned that it was very important for the app to be easy to use, such as by enabling automated self-monitoring through connected wireless devices instead of manual input of measures (eg, weight monitoring using a wireless scale connected to the app, physical activity monitoring using a fitness tracker). In addition, participants mentioned the app should be useful and provide value-added service, such as by enabling communication with clinicians and providing personalized feedback and education, instead of one-size-fits-all support.

## Discussion

### Principal Findings

This study identified the main facilitators and barriers to the use of mobile apps to support patients with heart failure self-management. The most important features mentioned by patients were communication between patients and clinicians, personalized feedback, automated self-monitoring, tracking of patient-reported measures, and mental health support. Participants suggested that those features could improve patient awareness about their condition, the ability to monitor their health status, and their understanding of the consequences of their behaviors, increasing their confidence and motivation to self-manage the disease. Lack of digital literacy and patients’ unwillingness to engage in a new strategy to manage their condition using an app were seen as the most relevant barriers. Clinician workload and remuneration were also mentioned by clinicians as potential barriers.

### Comparison With Prior Work

Our findings complement the results from 2 previous qualitative studies exploring patients’ perspectives on the use of mobile technologies for heart failure self-management [35,36]. Although these studies did not focus specifically on mobile apps, there were some common findings with our study, namely, the importance of ease of use and usefulness [35,36,51]. In these qualitative studies, as in ours, participants mentioned specific facilitators enhancing ease of use (eg, automated self-monitoring) [35] and usefulness (eg, communication with clinicians, personalized education) [36]. Both studies found that lack of digital literacy and willingness to use the technology were the most frequently mentioned barriers, in line with our results [35,36].

Our findings are consistent with reviews of relevant mobile app features for self-management of other chronic diseases [52,53]. Common facilitators from these studies included ease-of-use, personalized features (including changes in goals or needs depending on the stages of the disease), tracking of self-management tasks with visualization and analysis of trends and progress, and support from the clinicians (communication). One study also alerted to the need to track patients’ mental health, as suggested by some clinicians in our study [53]. Similar to these findings, the importance of integration with other platforms and devices and sharing information with other systems and supporting members (clinicians, family, caregivers) were also highlighted as important [52].

An interesting finding from our study was that clinicians highlighted the need for patient-reported measures and mental health tools to assess and address the psychological impact and emotional burden of heart failure [12]. Heart failure may affect patients’ mental health and has been associated with depression, anxiety, and cognitive disorders [12,54,55]. Mental health problems increase the risk of hospital readmissions, hamper treatment compliance, and affect patients’ quality of life [12,54,55]. Patient-reported outcomes can be collected through self-reported questionnaires assessing aspects such as general well-being, symptoms, functional impairment, or psychological status [56]. Patient-reported outcomes assessment might help clinicians to recognize and measure consistently the overall impact of heart failure and its treatment on patients’ lives, adding strategies to reduce or manage this impact (eg, adjusting diuretic doses, targeting depression management), and assess their response [56,57]. Mental health support via smartphone apps has shown promise in previous studies [58] but the evidence is lacking regarding their use by patients with heart failure.

### Strengths and Limitations

This study had several strengths and limitations. Interviewing different health care professional groups enabled us to gather perspectives on a variety of self-management aspects (eg, diet, physical activity, medication adherence, and mental health). Furthermore, recruitment of patients with and without experience using health apps enriched the understanding of the facilitators and barriers of adopting mobile apps for heart failure self-management. Main limitations of this study are as follows: first, the single practice setting and the lack of cardiologists among the clinicians interviewed; cardiologists could have enhanced the findings and raised new insights, and future studies should explore their perspectives. Second, the app and platform at use in the clinic were not specific for heart failure self-management, which may have limited the exploration of some features (eg, heart failure–specific education and alerts for early warning signs of decompensation). Third, the patients recruited to this study did not have previous experience with a heart failure–specific app, which allowed us to gather open-minded perspectives on potential facilitators and barriers and desirable features, but may have led to missed insights on some heart failure–specific app features. Fourth, the small sample did not allow comparison between patients using and not using the clinic’s app. Finally, because we did not collect electronic health record data, we could not characterize patients according to severity of heart failure.

### Future Directions

The design, evaluation, and implementation of apps to support heart failure self-management should focus on features enhancing their usefulness (eg, communication ability, personalized feedback, and education) and factors that facilitate ease of use (eg, automated self-monitoring). Chat-like functions may be an engaging way to support patients in-between clinical appointments, as well as may enable gathering information from patients on symptoms of heart failure decompensation. Other important health information to be monitored, such as weight and blood pressure, may be easily collected by wireless devices integrated with the app, to lessen self-management burden.

Finally, there seems to be a desire for features that help manage patients with heart failure not only physically or according to their tasks but also emotionally, providing mental health support. Although patient-reported outcomes have been largely used in clinical trials to evaluate quality of life, the adoption of patient-reported outcomes assessment in clinical care still needs development, and the use of mental health tools in mobile apps focused on heart failure is still largely unexplored.

### Conclusion

Mobile apps have potential in supporting heart failure self-management, particularly if including features desired by patients and clinicians, such as communication in-between visits, automated self-monitoring, and personalized feedback. These features should be considered in the future co-design of apps focused on the self-management of heart failure. While this qualitative study focused on the primary care setting, future studies should also involve cardiologists and patient caregivers.

## Appendix

Multimedia Appendix 1 Screenshots of the CareMonitor app.

Multimedia Appendix 2 Interview guide for patients.

Multimedia Appendix 3 Interview guide for clinicians.

Multimedia Appendix 4 COnsolidated criteria for REporting Qualitative research (COREQ) checklist.

## Abbreviations

COREQ: COnsolidated criteria for REporting Qualitative research

## Data Availability

Because of ethical and privacy issues, the data collected for this study are not available for sharing.
