# Supplementary material for: Use of Mobile Apps in Heart Failure Self-management: Qualitative Study Exploring the Patient and Primary Care Clinician Perspective
Source: JMIR Cardio. 2022 Apr 20;6(1):e33992. doi: 10.2196/33992 (PMC9069281; doi:10.2196/33992)

## Appendix 1: Screenshots of the CareMonitor app


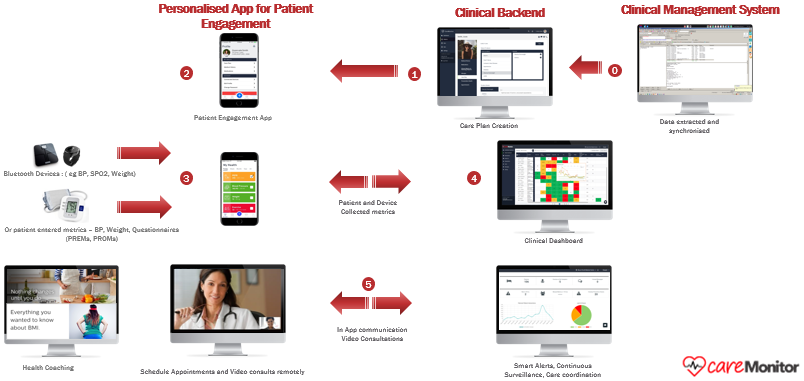


Screenshots of the CareMonitor patient app


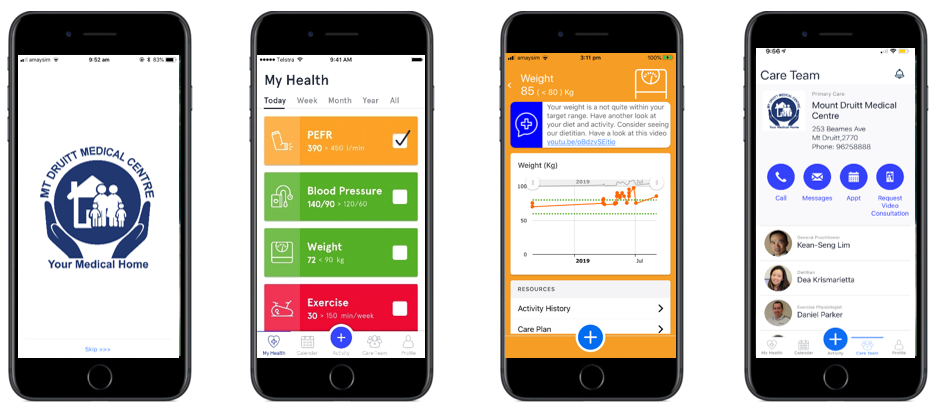


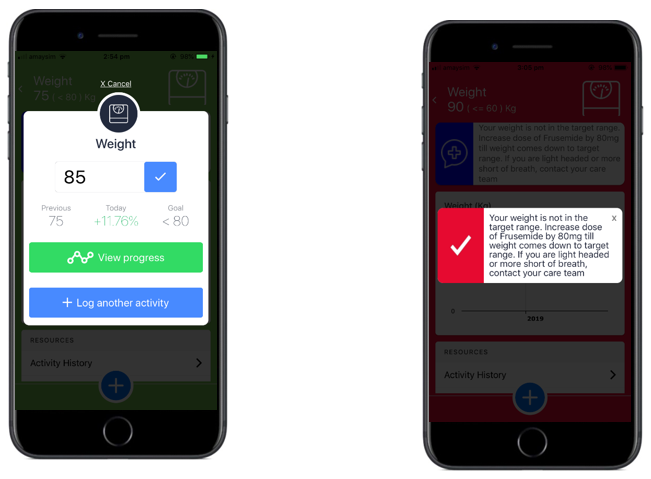


Screenshots of the CareMonitor clinician dashboard


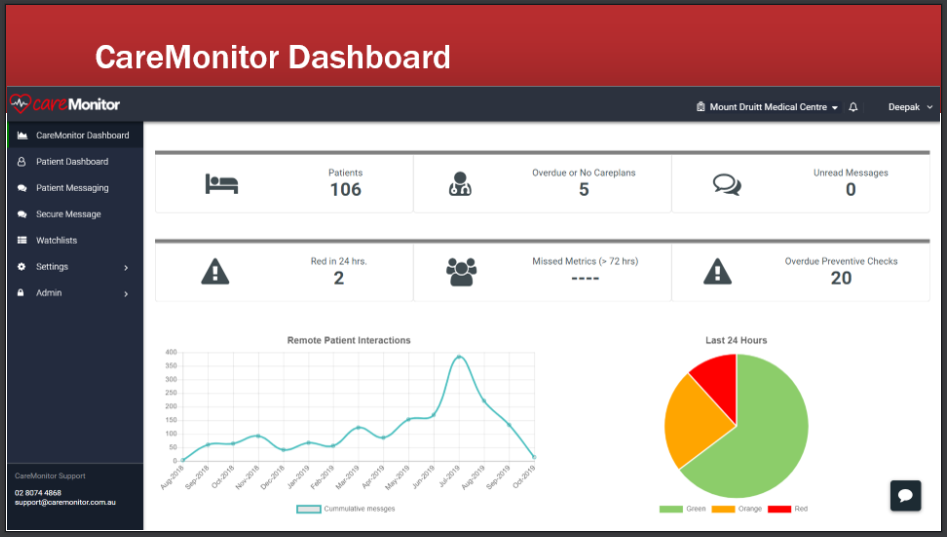


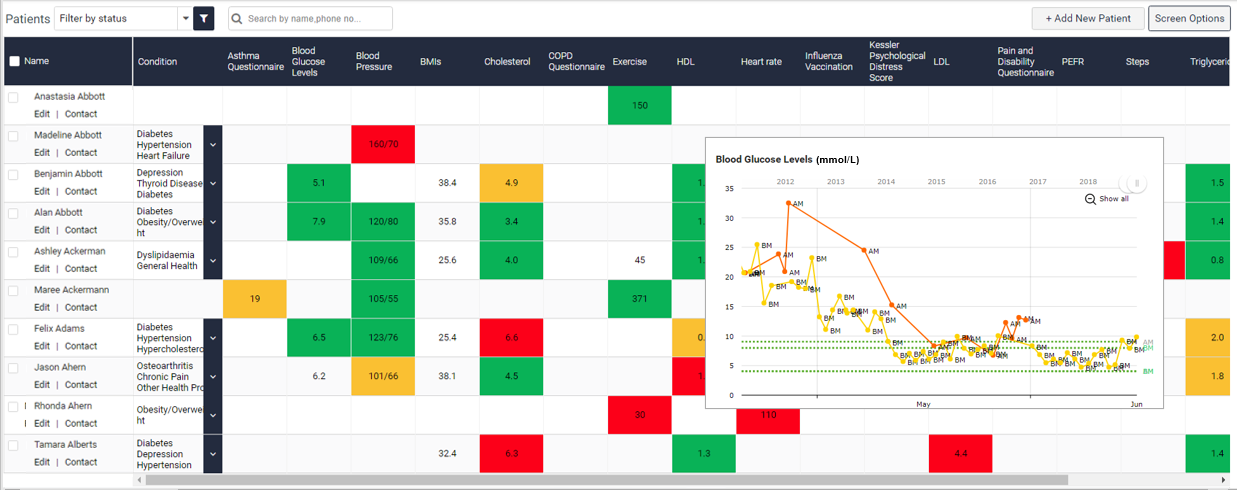

Supplement: Multimedia Appendix 1 [file cardio_v6i1e33992_app1.docx]
