# Supplementary material for: Use of Mobile Apps in Heart Failure Self-management: Qualitative Study Exploring the Patient and Primary Care Clinician Perspective
Source: JMIR Cardio. 2022 Apr 20;6(1):e33992. doi: 10.2196/33992 (PMC9069281; doi:10.2196/33992)
Supplement: Multimedia Appendix 2 [file cardio_v6i1e33992_app2.docx]

## Appendix 2: Interview guide for patients

Questionnaire

Age

Gender

Race / Ethnicity

Marital status

Education (High school or less / incomplete graduation/ graduate degree or higher)

Employed / Retired / Unemployed / Other

Disease information:

Year of HF diagnosis

Other health problems? Which ones?

Number and type of daily medication

Difficulty to read? (vision problems)

Any recent hospitalization? Why?

Self-management information:

Do you usually monitor your: (you can choose more than one)

- Weight
- Blood pressure
- Heart rate
- Liquid intake
- Symptoms
- None
- Other:

Heart Failure:

How do you deal with heart failure on a daily basis?

What are your main challenges? What do you think is more difficult to manage? (Prompts: Medication? Diet control? Physical activity?)

How do you remind yourself about your medication or appointments?

How do you control your liquid intake?

How often do you weigh yourself? Do you take any action if you gain weight?

How do you control your data? (Prompts: In a diary? On your phone or computer?)

What do you feel/what symptoms do you notice when your heart failure is worsening? (Prompts: Feet or legs swelling up? More fatigued than usual? Shortness of breath to sleep?)

What do you do when this happens?

What do you think it would help you to better manage your condition?

Mobile technology:

Do you own a mobile phone?

If yes, do you use it for:

- Internet access (smartphone)?
- Texting?
- App use? If yes, which kind? If not, why? (Prompts: Uninterested? Difficult? Useless?)

If you have used a smartphone app:

- Have you ever used a health application? If no, why? If yes, how was your experience?
- Did you find it easy or difficult?

Mobile apps for heart failure self-management:

Do you think that a mobile phone app could help you manage your condition?

Do you think you would need training or explanations to use it?

Suppose you can choose all the features for a new mobile app to help you to manage your heart failure…

- What kind of characteristics do you think would motivate you to use it? (Prompts: Ease-of-use? Cost? Voice? Big letters? Usefulness?)
- What do you think would be the most important tools? (Prompts: Record your data, such as weight, blood pressure, heart rate? Medication reminders? Appointments reminders? Information about the disease? Help you to know how much liquid or salt you have ingested?)

Do you use the CareMonitor app?

- If yes, what do you think are the positive and negative aspects of it. What do you find more or less useful? Is there any additional feature that you would like it to have?
- If not, why?
