# Supplementary material for: Use of Mobile Apps in Heart Failure Self-management: Qualitative Study Exploring the Patient and Primary Care Clinician Perspective
Source: JMIR Cardio. 2022 Apr 20;6(1):e33992. doi: 10.2196/33992 (PMC9069281; doi:10.2196/33992)
Supplement: Multimedia Appendix 3 [file cardio_v6i1e33992_app3.docx]

## Appendix 3: Interview guide for clinicians

Questionnaire

Age

Gender

Occupation

Years of practice at the clinic

Average of patients with heart failure you attend monthly

**Interview Guide – Clinicians**

Heart failure

What does a normal visit with patients with heart failure look like?

What kind of parameters do your patients normally collect as part of their self-management? (Prompts: How? In paper? Other?)

What do you think are the most challenging aspects of patients’ self-management? (Prompts: What information/data do they often forget when returning to a consultation? Weight? Liquid tracking? Medication?)

Mobile technology

What parameters would you like to monitor regularly from your patients, ideally?

In what situation would the monitored parameters make the care team intervene?

Do you believe that an app specifically designed for self-management of heart failure can help patients to actively manage their condition? If no, why? If yes, what do you think would be its most relevant features?

How do you think a shared platform between patients and clinicians should be to improve patients’ care? Most important features?

Do you use the CareMonitor app? If so, do you communicate with patients via the platform? What do you think are the advantages of the platform? What about disadvantages?

Do you think it is possible to have a shared platform without increasing the clinicians’ workload in a detrimental way?
